# Dual RNA-Seq Reveals Molecular Interactions Between *Alternaria alternata* and *Cucumis melo* During Postharvest Infection

**DOI:** 10.3390/foods15111876

**Published:** 2026-05-26

**Authors:** Yujia Bai, Xiangfeng Zheng, Bin Wu, Xiangyue Kong, Liuchun Du

**Affiliations:** 1 Institute of Food Science and Technology, Academy of Agricultural Sciences of Xinjiang Uyghur Autonomous Region, Urumqi 830091, China; xjuwubin0320@sina.com; 2 Xinjiang Key Laboratory of Processing and Preservation of Agricultural Products, Academy of Agricultural Sciences of Xinjiang Uyghur Autonomous Region, Urumqi 830091, China; 3 College of Food Science and Engineering, Yangzhou University, Yangzhou 225009, China; 4 College of Food Science and Pharmacy, Xinjiang Agricultural University, Urumqi 830052, China; 18346730352@163.com (X.K.); 13794104441@163.com (L.D.)

**Keywords:** interactions, *Alternaria alternata*, *Cucumis melo*

## Abstract

*Alternaria alternata* causes major postharvest losses in melons. This dual RNA-seq study analyzed transcriptomic dynamics in both *A. alternata* strain A2022 and melon during infection. Genomic analysis revealed two conditionally dispensable chromosomes (CDCs) containing host-specific toxin genes, including a T1PKS cluster for βenone biosynthesis. Fungal transcriptomics identified upregulated virulence pathways: MAPK signaling (18 genes), autophagy (17 genes), and lipid metabolism. Melon mounted a biphasic defense: early lignin deposition via phenylpropanoid biosynthesis (15 genes) and late jasmonic acid/MAPK-mediated responses. Competitive nutrient acquisition emerged, with fungal amino acid transporters opposing host nitrogen metabolism. These findings reveal *A. alternata*’s genomic plasticity and melon’s layered resistance, suggesting RNAi-based silencing of CDC virulence genes or breeding for enhanced lignin/jasmonate pathways as sustainable control strategies.

## 1. Introduction

Melons are economically vital worldwide, but Alternaria-induced blight, causing dark lesions and toxic metabolites, leads to significant postharvest losses [1]. The FAO estimates that fungal infections spoil 20–30% of global melon production, with *A. alternata* as the main pathogen. Fungicides are the primary control method, but their overuse has led to resistance and environmental concerns [2]. It is vital to clarify the molecular processes involved in Alternaria pathogenesis in melons and the host’s defense mechanisms to develop innovative, environmentally sustainable control strategies and breed disease-resistant strains.

*Alternaria alternata*, a globally distributed necrotrophic pathogen from the ascomycete genus Alternaria, significantly threatens agriculture by infecting over 100 plant species, including many fruits [3,4]. It uses various virulence factors, such as effector proteins, host-specific toxins (HSTs), non-host-specific toxins (nHSTs), and enzymes like cutinases and lipases, to invade and extract nutrients from hosts. Host-specific toxins are its main virulence tools [5,6]. These toxins target host cells and organelles like the Golgi apparatus, mitochondria, plasma membrane, and nucleus, disrupting key metabolic pathways such as ceramide and ATP synthesis [7]. Toxins affecting the plasma membrane cause increased potassium efflux. *Alternaria* pathogens produce host-specific toxins (HSTs) known as alternariol toxins, categorized into polyketides, cyclic peptides, aminopentenyl polyketides, and epoxy-decatrienoic acids [8]. These HSTs are often located on CDCs [9], which can be transferred between strains, leading to highly virulent strains with broad host ranges [10]. The genomes of 26 Alternaria species have been sequenced, aiding in the discovery and analysis of virulence factors [11]. Sequencing new pathogenic strains can reveal novel host-specific genes and insights into pathogenic mechanisms [12]. Recent studies highlight genome size and predicted gene content as key for identifying new virulence genes and their similarities to other pathogens [13].

The infection of fruits by *A. alternata* involves changes in gene expression and protein interactions between the pathogen and the host. During infection, the pathogen adapts to the host environment by modulating virulence-associated gene expression, while the host activates complex immune responses, including cell wall reinforcement, phytohormone signaling, reactive oxygen species accumulation, and defense-related metabolic pathways. Recent advances in transcriptomic technologies, particularly dual RNA-seq, have enabled simultaneous monitoring of host and pathogen gene expression during infection, providing a comprehensive understanding of the molecular dialog occurring in host–pathogen systems [14,15,16]. RNA sequencing is used to track these molecular changes and has been applied to study plant–pathogen interactions in crops like apple [17], citrus [18], grape [19], and soybean [20]. However, research on melon remains limited. Existing studies on melon under pathogen stress mainly rely on single transcriptome analyses and therefore lack insight into the dynamic interactions occurring between melon and *A. alternata*. Dual RNA-seq can simultaneously capture pathogen- and host-specific transcripts during infection, providing a comprehensive understanding of the biosynthetic pathways, metabolic processes, and gene expression networks involved in host–pathogen crosstalk [21], capturing pathogen-specific transcripts during infection and providing a comprehensive view of the interaction. This approach reveals biosynthetic and metabolic pathways and identifies gene expression profiles related to host–pathogen crosstalk. The molecular mechanisms of the interaction between melon and *A. alternata* are still unclear. Thus, dual RNA-seq of both organisms will enable simultaneous study of transcriptional changes during fungal infection and host resistance.

This study seeks to sequence the genome of the pathogenic strain *A. alternata* A2022 to understand its pathogenicity. We will use RNA-seq and dual RNA-seq to examine gene expression in melon during infection. By analyzing differentially expressed genes, we aim to identify crucial fungal virulence and host resistance genes, leading to a proposed molecular interaction model of *A. alternata*–melon infection. This research will deepen our understanding of the interaction between melon and *A. alternata*, reveal potential pathogenic mechanisms and host defense strategies, and provide valuable molecular resources for suppressing fungal pathogenicity and breeding disease-resistant melon cultivars.

## 2. Materials and Methods

### 2.1. Strains and Melon Used in This Study

*A. alternata* strain A2022 was isolated from decayed melon in China and identified through ITS rDNA analysis by the Xinjiang Agricultural Academy’s fruit and vegetable disease prevention team. It was cultured on potato dextrose agar (PDA: 200 g extract of boiled potatoes, 20 g glucose, 20 g agar, and 1 L distilled water) plates at 28 °C for 7 d and stored at 4 °C. Spores were scraped into centrifuge tubes and suspended in sterile distilled water. The spore concentration was determined using a hemocytometer and then adjusted to the desired concentration for the following experiment by using sterile distilled water. Jiashi melons, averaging 4.0 kg in mass, were collected from Xinjiang. The varieties were healthy and undamaged, with specific firmness and soluble solids content.

### 2.2. Perpetration of Genomic DNA of A. alternata Strain A2022

Genomic DNA from *A. alternata* strain A2022 was extracted using the Fungal Genomic Miniprep Kit (MesGen Biotech, Shanghai, China) as per the manufacturer’s instructions. DNA concentration was measured with the Quant-iT PicoGreen dsDNA Assay Kit, and integrity was checked via 1% agarose gel electrophoresis. The DNA was stored at −20 °C for future sequencing.

### 2.3. Genomic DNA Sequencing

Genomic DNA sequencing was conducted utilizing an integrated approach that combined second-generation (Illumina, San Diego, CA, USA) and third-generation (PacBio, Menlo Park, CA, USA) sequencing technologies. For the Illumina library preparation, the standard TruSeq Nano DNA LT Library Prep protocol, as outlined in the Illumina TruSeq DNA Sample Preparation Guide, was employed with the TruSeqTM DNA Sample Prep Kit. The workflow included DNA fragmentation using Covaris, end repair with End Repair Mix, 3′ end adenylation through A-tailing, adapter ligation, bead-based purification of ligation products, PCR amplification for library enrichment, and quantification via PicoGreen. Library quality control was conducted using the Agilent Bioanalyzer 2100 (Santa Clara, CA, USA) to verify fragment size distribution. Multiple DNA libraries were multiplexed, normalized to a concentration of 10 nM, pooled in equal volumes, and gradually diluted to 4–5 pM for sequencing. For the PacBio library preparation, the standard protocol was followed, which involved DNA shearing with g-TUBE, damage repair, end repair, hairpin adapter ligation, exonuclease digestion, and size selection using BluePippin (Beverly, MA, USA) to produce the final sequencing library, which was subsequently loaded for sequencing.

### 2.4. Genome Assembly and Bioinformatics Analysis

Filtered reads were assembled with Flye [22] and polished with Pilon [23] using Illumina data for accuracy. BWA [24] was used to map sequencing data back to the genome for evaluation. Genome completeness was assessed with BUSCO v2.0 [25] using the fungi_odb9 database. Gene prediction involved Genscan [26] for ab initio [27], GeMoMa [28] for homology-based, and Hisat2 [29] for transcriptome-guided methods using *Alternaria* sp. A2022 RNA-seq data. Unigenes were predicted with PASA [30] and integrated with EVM, followed by PASA refinement. Predicted gene sequences were annotated via BLAST against KOG [31], KEGG [32], and Nr databases [33], with GO annotation by Blast2GO and Pfam annotation using hmmer [34,35]. Enrichment analyses for KOG, KEGG pathways, and GO terms were performed. Predicted protein sequences were annotated using BLAST against databases like TCDB [36], PHI [37], CYPED [38], and DFVF [39]. Carbohydrate-active enzymes were identified with hmmer v1.2 [35] using the CAZy database [40]. SignalP 4.0 predicted signal peptides, and TMHMM screened for transmembrane helices to classify proteins with signal peptides but no transmembrane domains as secreted proteins [41].

### 2.5. RNA Extraction from the Sample

Healthy Jiashi melons were first rinsed with sterile distilled water, then immersed in 2% hydrogen peroxide solution for 30 s to surface-sterilize, followed by three rinses with sterile distilled water. After air-drying under aseptic conditions, melons were stored in a cool, dry aseptic environment prior to inoculation. Three equidistant puncture sites (3.5 mm diameter, 5 mm depth, 7 cm spacing) were created along the equatorial region of each melon. Each site was inoculated with 20 μL of *A. alternata* spore suspension (adjusted to 1.0 × 10^6^ spores/mL), while control groups received 20 μL of sterile distilled water per site. All inoculated melons were incubated at 28 ± 1 °C with 35–40% relative humidity. At 0, 4, and 10 days post-inoculation (dpi), six melons were randomly selected from each treatment group. For inoculated samples, diseased tissue was excised from the margin of the expanding lesion at the inoculation site; for control samples, identical-sized tissue was collected from the sterile water-treated sites. Total RNA was extracted from all collected tissues using a commercial plant RNA isolation kit following the manufacturer’s instructions (FastPure Universal Plant Total RNA Isolation Kit, Vazyme, Nanjing, China). The *A. alternata* in Jiash melons at 4 and 10 days are labeled JA04 and JA10, respectively. The Jiash melons treated with A2022 at 4 and 10 days were designated as JW04 and JW10, respectively. Untreated melon samples and *A. alternata* A2022 served as controls, named J00 and A2022. Each group had three replicates.

### 2.6. RNA-Seq Library Construction, Sequencing and Bioinformatics Analysis

RNA quality was assessed via gel electrophoresis, and concentration was measured with an SMA1000 ultramicro spectrophotometer (Merinton, Beijing, China). Samples were sent to Gene-pioneer Biotechnologies for library construction and transcriptome sequencing. Clean reads were obtained by filtering out low-quality reads, poly-N, and adapters. HISAT2 [29] software aligned these reads to the *A. alternata* reference genome. StringTie software (v2.2.1, Johns Hopkins University, Baltimore, MD, USA) then assembled the reads for further analysis. TopHat v2.0.12 was used to align paired-end clean reads. This process created a splice junction database. Gene expression levels were calculated with the FPKM metric using HTSeq v0.6.1 [42]. Differentially expressed genes (DEGs) with |log_2_ fold change| ≥ 1 and *p* < 0.005 were identified using the DEGSeq R package (version 1.20.0) [43].

### 2.7. Gene Ontology and Kyoto Encyclopedia of Genes and Genome Pathway

The enrichment analysis of DEGs was conducted using the GOseq R package (v1.48.0, Bioconductor) and the KOBAS software (v3.0, http://kobas.cbi.pku.edu.cn/, Peking University, Beijing, China; Chinese Academy of Sciences, Beijing, China), as described by Alexa et al. [44] and Xie et al. [45]. Significantly enrichment analysis was determined based on a corrected *p*-value (*p* < 0.05).

### 2.8. Determination of Relative Expression Level of Some Key Genes

The RNA samples utilized for the assessment of gene expression levels were prepared as outlined in Section 2.3. Subsequently, complementary DNA (cDNA) was synthesized from 1 μg of RNA using the PrimeScript RT reagent kit with gDNA Eraser (TAKARA, Tokyo, Japan), following the manufacturer’s protocol. Real-time quantitative polymerase chain reaction (RT-qPCR) was conducted as described the SYBR Premix Ex Taq II Tli RNaseH Plus (TAKARA, Tokyo, Japan) on a Q1000 Real-Time PCR System (LongGene, Hangzhou, China). Each sample was analyzed in triplicate.

### 2.9. Statistical Analysis

The data were presented as the mean ± standard deviation, with error bars representing the standard deviation in the plots. An analysis of the data was conducted using ANOVA in SPSS 26.0, and Duncan’s multiple range test was applied with a significance level (α-level) of 0.05, corresponding to a *p*-value threshold of <0.05 to determine statistically significant differences.

## 3. Results

### 3.1. Genomic DNA Sequence of A2022

PacBio and Illumina NovaSeq technologies were employed to produce 6.28 Gb of paired-end short sequence reads. The assembly of *A. alternata* strain A2022 resulted in a total size of 34.79 Mb, with a GC content of 50.93% and a contig N50 of 3.33 Mb. The finalized assembly of strain A2022 comprised 10 contigs, culminating in a total genome size of 34,792,429 base pairs (Table 1). The completeness of the genome assembly was assessed using benchmarking universal single-copy ortholog (BUSCO) version 5.0b with the ‘fungi_odb10’ library, revealing a genome completeness (BUSCO complete + partial) of 98.9% for *A. alternata* strain A2022. Additional genomic statistics for strain A2022 are presented in Table 1. The genome encompasses 13,672 protein-coding genes. *A. alternata* strain A2022 consists of 10 essential chromosomes and two conditional CDCs, corroborating the experimental evidence obtained through pulsed-field gel electrophoresis (GenBank accession numbers CP061875.1 to CP061886.1).

### 3.2. Annotation of the Proteins

The protein annotation results are in Table 2. Using Protein-Protein BLAST v2.6.0 against the Swiss-Prot database with specific parameters, 7523 genes (55.18% of the proteome) were functionally annotated. The dbCAN v6.0 database identified 850 CAZYmes (11.30% of the proteome) using hmmscan, including various enzyme types. Of these, 24 genes are linked to fibroin degradation, 30 to hemicellulose, 41 to pectin, and 11 to lignin. Additionally, 540 genes were annotated in the Transporter Classification Database (TCDB). Pathogenicity genes were predicted using BLASTP against the PHI database, identifying 3574 potential PHI genes. The results of the protein annotation was shown in Table 2. The proteins were functionally annotated by using Protein-Protein BLAST v2.6.0 against the Swiss-Prot database with the parameters ‘–evalue 1e-5–max_target_seqs 20’, which resulted in a best-hit description for 7523 genes representing 55.18% of the proteome. The dbCAN v6.0 database was used to predict CAZYmes by using hmmscan and showed 850 CAZYmes (11.30% of the proteome), which included 291 glycosyl hydrolases, 154 carbohydrate esterases, 109 glycoside transferases, 111 carbohydrate-binding modules, 160 auxiliary activities, and 25 polysaccharide lyases. Among these 850 genes, 24 genes were involved in fibroin degradation, 30 genes in hemicellulose degradation, 41 genes in pectin degradation and 11 genes in lignin degradation. A total of 540 genes were annotated to the Transporter Classification Database (TCDB). The pathogenicity genes were predicted by using BLASTP against the Pathogen–Host Interaction (PHI) database with a strict threshold of Match ^3^ 50% and Identity ^3^ 50%, which identified 3574 putative PHI genes. There are 101 Increased Virulence genes and 122 Lethal genes linked to Alternaria SPP pathogenicity. SignalP v5.0 analysis of *A. alternata* A2022 identified 1162 proteins with secretion signals, 85 of which degrade cell walls. Secondary metabolites (SMs) are crucial for fungal survival, with antiSMASH 5.0 predicting 30 SM gene clusters, including five NRPS, five T1 PKS, four tepene, four NRPS-like, one fungal-RiPP, eight fungal-RiPP-like, one T3 PKS, one NAPAA, and one isocyanide-nrp. A T1PKS from CDC_1 synthesizes a Betaenone compound, involving seven genes.

### 3.3. The Genes That Exhibit Differential Expression in A2022 When Cultivated on Melon at Various Time Points

**Figure 1:**
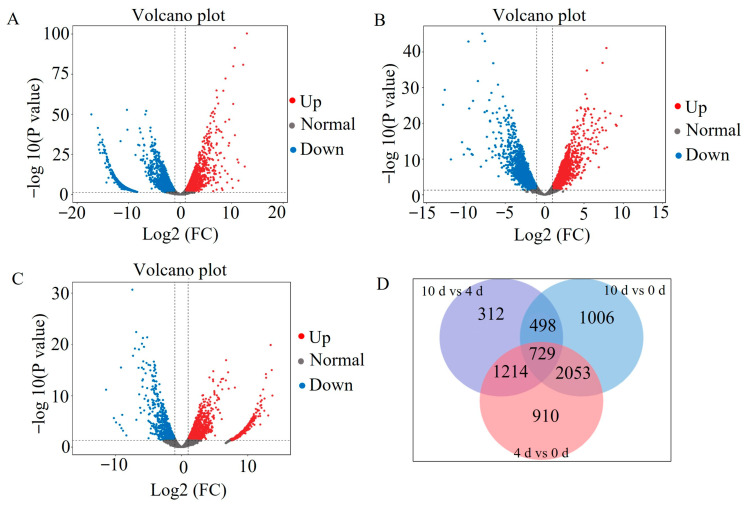
Overview of differential expression analysis across three pairwise comparisons. (**A**–**C**) Volcano plots illustrating the distribution of differentially expressed features in three independent comparisons. The x-axis represents log_2_-transformed fold change (FC), and the y-axis represents −log_10_-transformed *p* values. Red points denote significantly upregulated features, blue points denote significantly downregulated features, and gray points denote non-significant features. (**D**) Venn diagram showing the overlap of differentially expressed features among the three comparisons (10 d vs. 0 d, 4 d vs. 0 d, and 10 d vs. 4 d). Numbers in each region indicate the count of unique or shared differentially expressed features.

Twelve samples underwent RNA-seq, yielding 153.90 Gb of clean data, with each sample contributing 11.96 Gb. The GC content ranged from 43.06% to 52.20%, and over 92.55% of bases had a Q30 score, indicating high sequencing quality. Supplementary Tables S1 and S2 show high-efficiency alignment of mapped and uniquely mapped reads. Comparing *A. alternata* in melon at 4 and 10 days to 0 h, 4905 DEGs were identified in A2022 at 4 days, with 1760 significantly upregulated and 3145 significantly downregulated (Log_2_ Fold Change ≥1 or ≤−1, *p* < 0.05) (Figure 1A). In A2022, 4285 genes showed significant differential expression at 10 days compared to 0 days, with 2018 upregulated and 2267 downregulated (Figure 1B). Between 10 and 4 days, 2752 genes exhibited differential expression, including 1937 up regulated and 815 down regulated (Figure 1C). Among the DEGs, there are 729 genes that are shared by all three groups (Figure 1D).

### 3.4. GO and KEGG Analysis the DEGs of A2022

**Figure 2:**
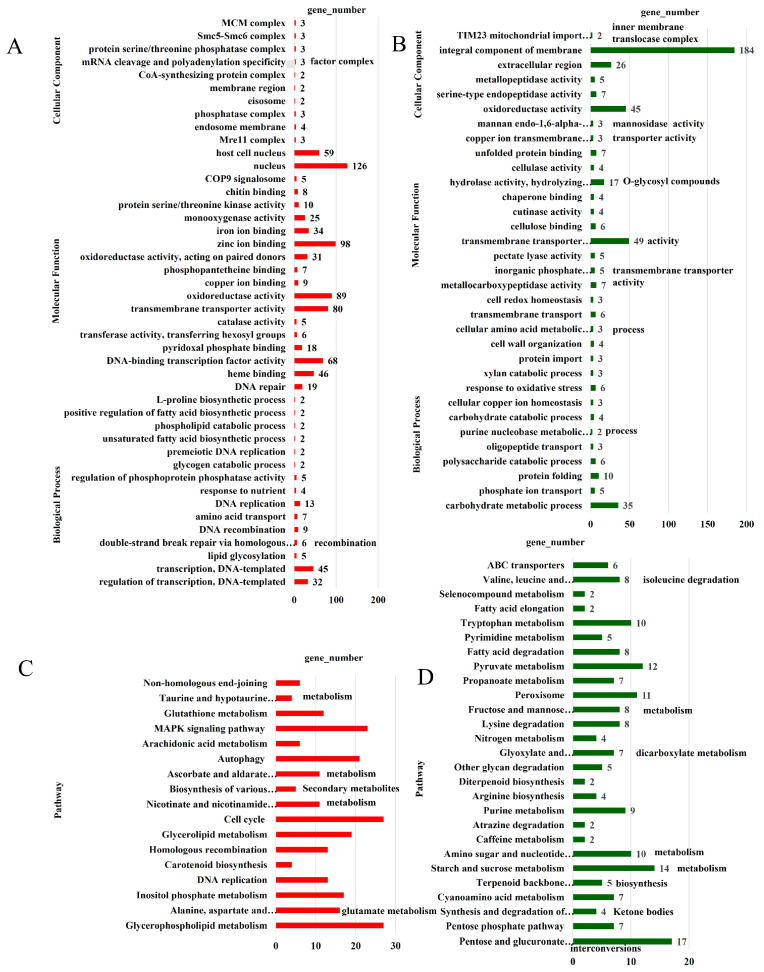
GO and KEGG enrichment analyses of differentially expressed genes (DEGs) in A2022 between days 4 and 10 post-infection.(**A**,**B**) Gene Ontology (GO) enrichment analysis of upregulated DEGs (**A**) and downregulated DEGs (**B**), categorized into three main domains: Cellular Component (top), Molecular Function (middle), and Biological Process (bottom). Bar length represents gene number; red bars indicate upregulated DEGs, green bars indicate downregulated DEGs. Top enriched terms are labeled. (**C**,**D**) Kyoto Encyclopedia of Genes and Genomes (KEGG) pathway enrichment analysis of upregulated DEGs (**C**, red bars) and downregulated DEGs (**D**, green bars). Pathway names are shown on the left; bar length reflects gene count.

Between days 4 and 10, a critical phase for *A. alternata* infection, an analysis of differentially expressed genes (DEGs) was conducted. GO enrichment analysis categorized DEGs into cellular components, biological processes, and molecular functions (Figure 2). The top enriched level-2 categories for upregulated DEGs at day 10 included the COP9 signalosome, nucleus, and host cell nucleus. Key biological processes with upregulated DEGs were regulation of transcription, lipid glycosylation, double-strand break repair via homologous recombination, DNA recombination, amino acid transport, DNA replication, and DNA repair (Figure 2A). In biological processes, the most enriched categories with down-regulated DEGs are carbohydrate metabolism (35 DEGs), protein folding (10), polysaccharide catabolism (six), response to oxidative stress (six), and transmembrane transport (six). In molecular function, transmembrane transporter activity is most enriched with 49 down-regulated DEGs. Other notable categories include oxidoreductase activity (45 DEGs), hydrolase activity (17), and several others with fewer DEGs (Figure 2B). KEGG analysis of DEGs identified the most enriched pathways as glycerophospholipid metabolism, alanine, aspartate and glutamate metabolism, inositol phosphate metabolism, DNA replication, carotenoid biosynthesis, homologous recombination, and glycerolipid metabolism. These pathways were linked to 27, 16, 17, 13, 4, 13, and 19 upregulated DEGs, respectively (Figure 2C), aiding A2022’s nutrient acquisition and growth in melon for a competitive edge. KEGG analysis showed Pentose and glucuronate interconversions had the highest enrichment with 17 downregulated DEGs. Other enriched pathways included starch and sucrose metabolism, amino sugar and nucleotide sugar metabolism, peroxisome, pyruvate metabolism, and tryptophan metabolism with 14, 10, 11, 12, and 10 downregulated DEGs, respectively (Figure 2D).

### 3.5. The Pathways and Genes Implicated in Conferring a Competitive to A2022

**Figure 3:**
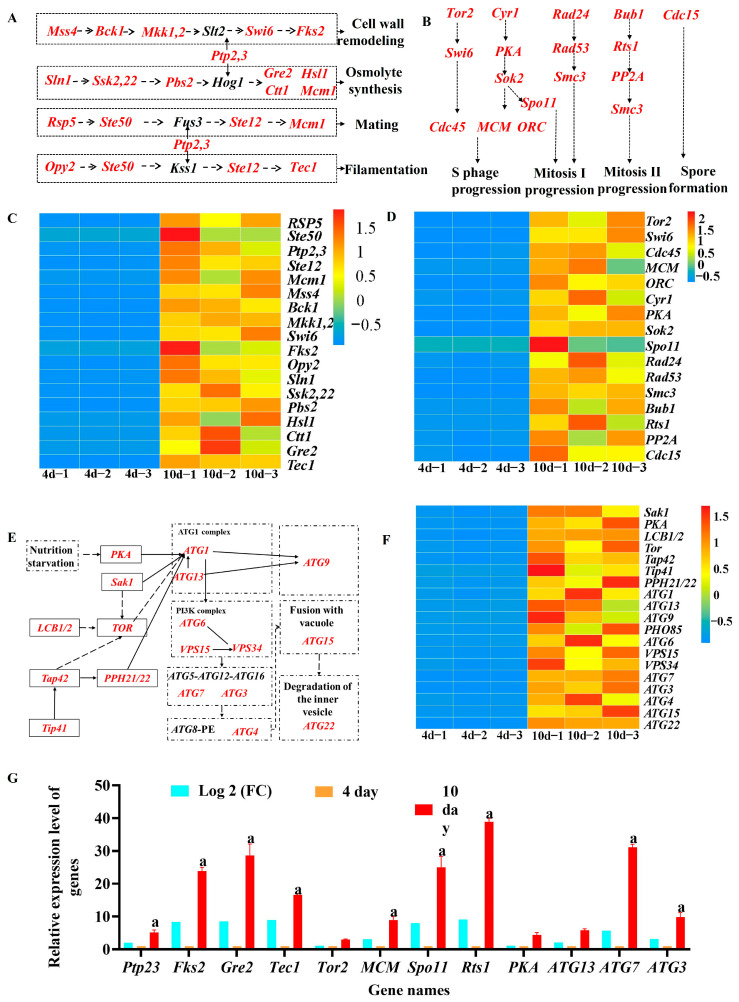
Expression and functional analysis of key pathways in *A. alternata* A2022 during infection (4–10 days). (**A**,**B**) Schematics of mycelial growth (**A**) and meiosis/spore formation (**B**) pathways, with upregulated genes highlighted in red. (**C**,**D**) Heatmaps of DEGs in mycelial growth (**C**) and meiosis (**D**), showing upregulation at day 10. (**E**,**F**) Autophagy pathway schematic (**E**) and heatmap of autophagy-related DEGs. (**G**) RT-qPCR validation of key genes, confirming consistency between transcriptomic and qPCR expression trends. Different lowercase letters above the bars indicate significant differences between 4 and 10 days (*p* < 0.05). Bars sharing the same letter are not significantly different.

The GO and KEGG analysis examined genes involved in mycelial growth, the cell cycle, and meiosis, focusing on expression changes from 4 to 10 days. Figure 3A shows 18 upregulated DEGs linked to mycelial growth, with their expression levels over 4–10 days in Figure 3C. These genes were significantly upregulated at 10 days, indicating the mycelial growth pathway’s role in A2022 infection. Additionally, 17 genes were upregulated during meiosis (Figure 3B,D). The upregulation of 17 meiosis-related genes at 10 dpi is associated with the pathogen’s preparation for conidiation, which supports secondary infection of melon tissues and ensures transmission to new hosts after successful colonization, representing an adaptive reproductive strategy under host infection conditions. Autophagy is key for intracellular protein degradation and supports cellular growth. In this study, 17 genes linked to the TORC signaling pathway and various complexes (ATG1, ATG9, PtdIns 3-kinase, and ATG5-ATG12-ATG16) were significantly upregulated, playing a vital role in autophagy regulation in A2022 (Figure 3E,F). Additionally, two differentially expressed genes in the autophagy pathway, involved in vesicle degradation and amino acid recycling, were also upregulated. This gene upregulation enhanced the autophagy process and accelerated inner vesicle degradation in A2022 in melon, as shown in Figure 3E. RT-qPCR detected expression levels of key genes related to mycelial growth, meiosis, and autophagy, which aligned closely with transcriptome data (Figure 3G).

### 3.6. The Genes in Melon Exhibit Differential Expression Following Treatment with A2022

**Figure 4:**
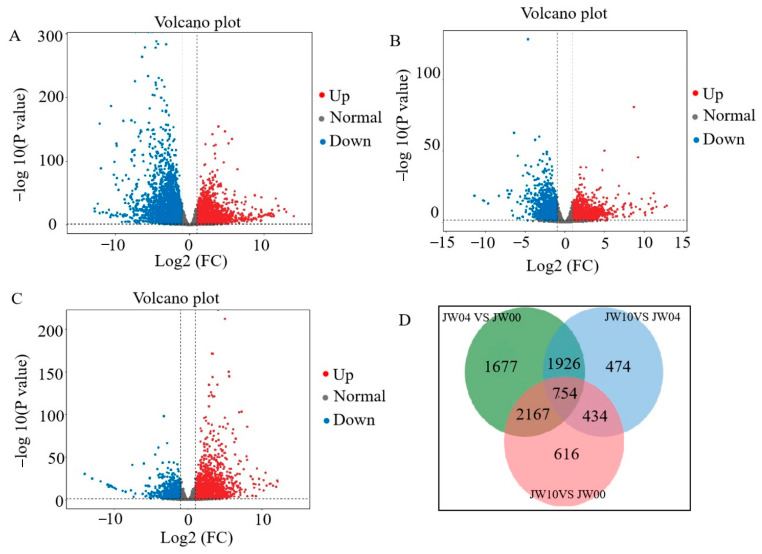
Differential gene expression profiles in melon following A2022 treatment.(**A**) Volcano plot of differentially expressed genes (DEGs) between JW04 (treated for 4 days) and J00 (untreated control). Red dots: up-regulated genes; blue dots: down-regulated genes; gray dots: non-significant genes. |Log_2_(FC)| > 1 and adjusted *p*-value < 0.05. (**B**) Volcano plot of DEGs between JW10 (treated for 10 days) and J00. Same color coding as in (**A**). (**C**) Volcano plot comparing DEGs between JW10 and JW04. (**D**) Venn diagram showing the overlap of DEGs among the three pairwise comparisons: JW04 vs. J00 (green), JW10 vs. JW04 (blue), and JW10 vs. J00 (pink). Numbers indicate the count of unique or shared DEGs.

Melon treated with A2022 (JW04) for 4 days showed 6907 differentially expressed genes (DEGs) compared to untreated melon (J00), with 3270 up-regulated and 3637 down-regulated genes (Figure 4A). After 10 days (JW10) of treatment, 4192 DEGs were identified, including 2278 up-regulated and 1914 down-regulated genes (Figure 4B). Comparing 10-day (JW10) to 4-day (JW04) treatments revealed 3826 DEGs, with 2318 up-regulated and 1508 down-regulated genes (Figure 4C). Among the DEGs, there are 754 genes that are shared by all three groups (Figure 4D).

### 3.7. GO and KEGG Analysis the DEGs of Melon in Response to A2022

**Figure 5:**
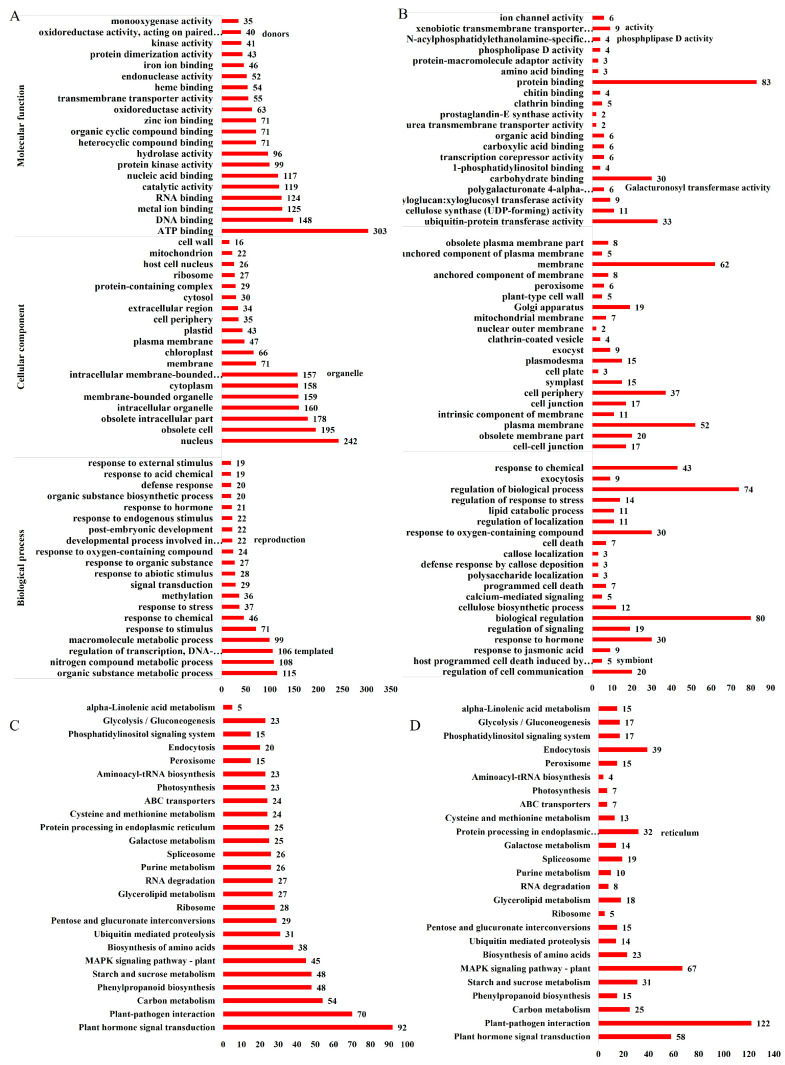
GO and KEGG enrichment analyses of differentially expressed genes (DEGs) in *Melon* during the critical infection phase between days 4 and 10. (**A**) GO enrichment of upregulated DEGs; (**B**) GO enrichment of downregulated DEGs; (**C**) KEGG pathway enrichment of upregulated DEGs. (**D**) KEGG pathway enrichment of downregulated DEGs.

The analysis focused on differentially expressed genes (DEGs) in melon treated with A2022, particularly between days 0–4 and 4–10, due to the observed fastest growth rate at 0 to 10 days. GO enrichment analysis was performed on DEGs upregulated between days 4–0 and 10–4, categorizing them into cellular components, biological processes, and molecular functions. The top level-2 categories with the highest enrichment scores are shown in Figure 5. Notably, within cellular components, integral components of membrane (697), nuclei (242), obsolete cells (195), and obsolete intracellular parts (178) were prominent (Figure 5A). Biological processes included organic substance metabolic process (115 DEGs), nitrogen compound metabolic process (108), regulation of transcription (106), DNA-templated macromolecule metabolic process (99), response to stimulus (71), response to chemical (46), response to stress (37), and methylation (36) (Figure 5A). The molecular function analysis shows that protein binding is the most enriched category, with 49 down-regulated DEGs. Other enriched categories include ubiquitin-protein transferase activity, cellulose synthase activity, and carbohydrate binding (Figure 5B). KEGG analysis of up-regulated DEGs between 4 and 0 days or 10 and 4 days identified key pathways involved in the defense response to A2022, such as plant hormone signal transduction, plant–pathogen interaction, and carbon metabolism. The pathways were linked to 92, 70, 54, 48, 48, 45, 38, and 31 upregulated DEGs, respectively (Figure 5C). KEGG analysis of upregulated DEGs at 10 days identified the most enriched pathways as plant hormone signal transduction (58), plant–pathogen interaction (122), starch and sucrose metabolism (31), MAPK signaling pathway-plant (67), protein processing in the endoplasmic reticulum (32), and Endocytosis (39) (Figure 5D).

### 3.8. The Pathways and Genes Linked to the Induced Defense Response of Melon Against Pathogens

**Figure 6:**
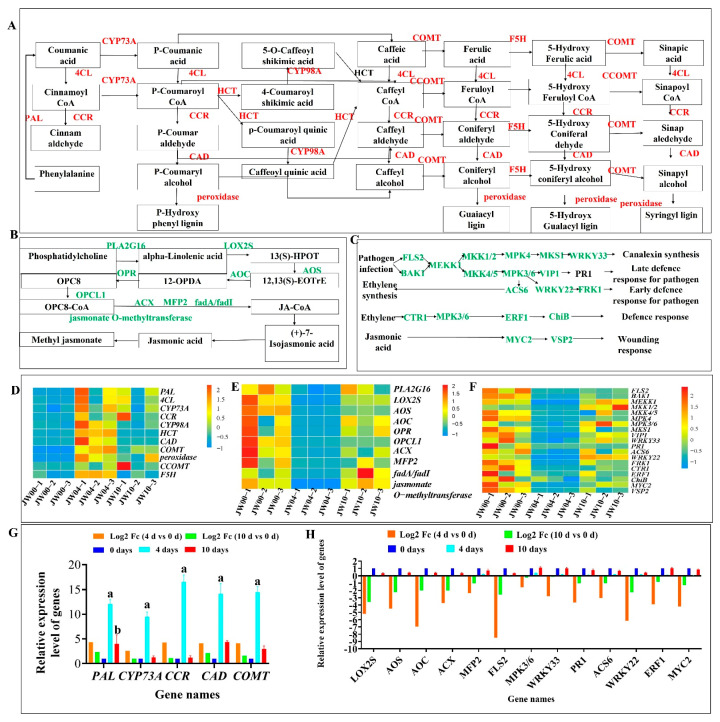
Defense pathway gene expression in melon during *A. alternata* A2022 infection. (**A**–**C**) Schematics of phenylpropanoid biosynthesis (**A**), α-linolenic acid/JA biosynthesis (**B**), and MAPK signaling (**C**) pathways. (**D**–**F**) Heatmaps showing expression profiles of pathway-related DEGs at 0, 4, and 10 days post-inoculation. In the heatmaps, colors represent the log2 fold change (Log2 Fc) of gene expression: red indicates upregulation (positive values), and blue indicates downregulation (negative values); color intensity corresponds to the magnitude of change. Phenylpropanoid pathway genes were upregulated early (4 dpi) and sustained through 10 dpi, while JA and MAPK pathway genes were induced primarily at 10 dpi. (**G**,**H**) RT-qPCR validation of key genes, confirming consistency between transcriptomic and qPCR expression trends. In the bar charts, gene expression levels are represented by colored bars: orange for 0 days, green for 4 days, and red for 10 days post-inoculation. Different lowercase letters above the bars indicate significant differences among 0, 4 and 10 days (*p* < 0.05). Bars sharing the same letter are not significantly different.

Gene expression analysis primarily focused on comparing inoculated melon tissues collected at different infection stages (0, 4, and 10 dpi) following *A. alternata* A2022 inoculation. The study found that A2022 treatment in melons enhances disease resistance by upregulating genes in the phenylpropanoid biosynthesis pathway, particularly on the fourth day. This includes increased expression of phenylalanine ammonia-lyase (PAL) and other key enzymes, boosting lignin synthesis and strengthening the plant’s defense against pathogens (Figure 6A). Melon enhances its defense by activating the phenylpropanoid biosynthesis pathway, with significant upregulation of 15 genes lasting until the 10th day (Figure 6D). On the 4 th day, only five genes in the alpha-Linolenic acid metabolism pathway were upregulated, but by the 10 th day, 15 genes showed significant upregulation (Figure 6B). This pathway is crucial for synthesizing jasmonic acid, a plant defense hormone. The key regulatory gene ACX was notably upregulated on the 10th day, indicating that the jasmonic acid pathway plays a significant role in melon’s resistance to pathogens in the later infection stage (Figure 6E). The study found that the MAPK pathway, like the jasmonic acid pathway, was not activated on day 4, with genes such as MEKK1, MKK1/2, MPK4, and MPK3/6 significantly downregulated (Figure 6C). However, by day 10, these genes were significantly upregulated, indicating MAPK pathway activation to combat pathogen infection (Figure 6F). RT-qPCR detected expression levels of key genes related to above pathway, which aligned closely with transcriptome data (Figure 6G,H).

## 4. Discussion

The study’s genomic and transcriptomic data reveal the molecular dynamics between *A. alternata* A2022 and *C. melo*. It identifies two CDCs with host-specific toxin clusters, like the betaenone-producing T1PKS, highlighting genomic plasticity’s role in pathogenic evolution. These findings support previous studies showing that CDCs in Alternaria species aid in virulence gene transfer for host adaptation [8,9]). The upregulation of 18 MAPK signaling pathway genes during infection emphasizes their importance in fungal virulence, aligning with research on other necrotrophic fungi like *Botrytis cinerea*, where MAPK cascades manage responses to host defenses and environmental stresses [46].

*A. alternata* A2022 uses autophagy and lipid metabolism to adapt to nutrient scarcity in its host. Upregulation of autophagy genes aids growth under limited resources, while lipid metabolism breaks down host membranes for carbon. This mirrors findings in *Fusarium graminearum*, where lipid use is vital for growth [47]. As infection progresses, the pathogen shifts from carbohydrate breakdown to toxin-driven necrotrophy, enhancing its competitiveness by focusing on amino acid metabolism for rapid proliferation in the melon. *C. melo* uses a two-phase defense against *A. alternata*. The enrichment of α-linolenic acid metabolism and MAPK signaling pathways highlights their important roles in melon defense against *A. alternata*. The α-linolenic acid pathway serves as a major source of jasmonic acid biosynthesis, which is essential for resistance against necrotrophic pathogens. MAPK signaling further mediates rapid immune signal transduction and defense gene activation [48,49]. Interestingly, several genes associated with these pathways exhibited relatively high expression levels at 0 dpi compared with 4 dpi. This pattern may reflect constitutive basal defense activity in healthy fruit tissues prior to visible infection. The transient downregulation at 4 dpi may indicate early suppression of host immune responses by *A. alternata* during the initial colonization stage, followed by subsequent reactivation of defense pathways as infection progressed. Initially, at 4 dpi, it also activates the phenylpropanoid pathway, involving enzymes like PAL, 4CL, and CAD, to deposit lignin and block fungal entry. This lignin buildup, also seen in other crops like tomatoes, strengthens cell walls against pathogens. By 10 dpi, the plant switches to a jasmonic acid-based defense, upregulating genes in the α-linolenic acid pathway, particularly ACX, to produce PR proteins and antimicrobials, similar to responses in Arabidopsis against *Alternaria brassicicola* [50]. At 10 dpi, the delayed activation of the MAPK signaling pathway enhances JA-mediated defenses and coordinates ROS production, leading to a hypersensitive response that limits pathogen spread. This timing separates lignin-based and JA-dependent defenses, optimizing resource use and balancing growth with immunity, similar to other plant–pathogen systems [51].

The dual RNA-seq analysis suggests that the interaction between *A. alternata* and *C. melo* is characterized by intense nutritional competition, particularly for nitrogen resources. The significant upregulation of fungal amino acid transporters together with the enrichment of host nitrogen compound metabolic processes indicates that both organisms actively regulate amino acid flux during infection. Nitrogen acquisition is increasingly recognized as a critical determinant of fungal pathogenicity and host resistance in plant–pathogen interactions [52,53]. Previous studies have shown that necrotrophic fungi can manipulate host nitrogen metabolism and amino acid transport systems to promote fungal proliferation and virulence, while plants simultaneously activate defense-associated nitrogen remobilization pathways to restrict nutrient availability to invading pathogens [54,55]. In our study, the induction of amino acid transport-related genes in *A. alternata* likely enhances fungal nutrient uptake under host-imposed nutrient limitation, whereas the activation of nitrogen metabolism pathways in melon may reflect a host strategy to rebalance metabolic resources and support defense responses. Similar nutrient competition mechanisms have been reported in several fungal pathosystems, where efficient nitrogen utilization directly influences infection success and disease progression [56,57]. These findings collectively indicate that the outcome of the *A. alternata*–*C. melo* interaction may largely depend on the ability of either organism to control nutrient acquisition and amino acid metabolism during infection. Fungal enzymes degrade host membranes, while the host produces protective waxes and suberin. Melon’s NLR-like resistance genes, similar to those for powdery mildew resistance, suggest shared defense strategies against fungi [58]. *A. alternata* uses effectors like LysM proteins to evade host immunity by binding chitin oligomers, a tactic also seen in Colletotrichum [59]. These insights reveal the complexity of host–pathogen dynamics and the pathogen’s evasion tactics.

The mechanistic model outlines the interaction between *A. alternata* and melon during infection. Early on (0–4 d), the fungus uses HSTs and MAPK signaling to damage host cells, while melon forms lignin barriers. Later (4–10 d), fungal growth is supported by autophagy and lipid metabolism, countered by melon’s JA/MAPK defenses. This model suggests targeting pathogen vulnerabilities like CDC-localized virulence genes with RNAi or CRISPR-Cas9 to reduce pathogenicity [60]. For melon, enhancing resistance through pyramiding lignin biosynthesis and JA pathway alleles, as shown in transgenic rice [61], is promising. Future work should validate these genes in transgenic melon and explore microbiome-mediated defense priming to boost resistance, as indicated by studies on plant microbiota [62].

## 5. Conclusions

This study combined whole-genome sequencing and dual RNA-seq analysis to investigate the molecular interaction between *A. alternata* A2022 and *C. melo* during postharvest infection. The results reveal the dynamic balance between fungal virulence and host defense responses and provide new insight into the pathogenic mechanisms underlying melon decay. A major novelty of this work is the simultaneous characterization of pathogen and host transcriptional responses during infection, together with the identification of CDCs carrying host-specific toxin-associated genes. The study further demonstrates that *A. alternata* adapts to the host environment through MAPK signaling, autophagy, lipid metabolism, and nutrient acquisition strategies, whereas melon activates sequential lignin biosynthesis and jasmonic acid/MAPK-mediated defense pathways. Importantly, the transcriptomic signatures identified in this study, including toxin biosynthesis genes, amino acid transporters, and CDC-associated virulence factors, may provide potential molecular markers for rapid pathogen detection and monitoring in postharvest food safety systems. These findings are consistent with current molecular approaches used in foodborne fungal pathogen identification and early-warning surveillance, particularly genome-assisted detection and transcriptome-based pathogenicity assessment. However, several limitations should be acknowledged. Functional validation of candidate virulence genes and host resistance genes was not conducted in this study, and the interaction was examined using only one fungal strain and one melon cultivar under controlled experimental conditions. In addition, transcriptomic data alone cannot fully capture post-transcriptional regulation or metabolite-level interactions during infection. Future studies integrating gene functional analysis, metabolomics, proteomics, and microbiome profiling will provide a more comprehensive understanding of the *A. alternata*–melon interaction and facilitate the development of sustainable disease management strategies.

## Figures and Tables

**Table 1:** Summary of the genome assembly and annotation statistics of *A. alternata* A2022.

Strain	A2022
Genome size (Mb)	34.79
GC content (%)	50.93%
Contig N50 (Mb)	3.33
Contig N90 (Mb)	2.04
Number of contigs	10
genome completeness (%) ^a^	98.9%
Number of protein-coding genes	13,672
Number of chromosomes	10
Number of conditionally dispensable chromosomes (CDCs)	2

^a^: Benchmarking universal single-copy ortholog BUSCO complete + partial.

**Table 2:** Summary of the protein annotation of *A. alternata* A2022.

Proteins Annotation	Function	Number
Swiss-Prot database		7523
CAZYmes	glycosyl hydrolases	291
	carbohydrate esterases	154
	glycoside transferases	109
	carbohydrate-binding modules	111
	auxiliary activities	160
	polysaccharide lyases	25
	fibroin degradation	24
	hemicellulose degradation	30
	pectin degradation	41
	lignin degradation	11
Transporter Classification Database		540
Pathogen–Host Interaction	Unaffected pathogenicity	1389
	Reduced virulence	1339
	Loss of pathogenicity	258
	Lethal	122
	Increased Virulence	101
	Effector	43
	Chemistry target	23
	Enhanced antagonism	1
SignalP v5.0 analysis	Degrading cell wall	85
Secondary metabolites	nonribosomal peptides synthetase [NRPS]	5
	type 1 [T1] polyketide synthase [PKS]	5
	terpene	4
	NRPS-like	4
	fungal-RiPP	1
	fungal-RiPP like	8
	T3 PKS	1
	NAPAA	1
	isocyanide-nrp.	1

## Data Availability

The original contributions presented in this study are included in the article/Supplementary Materials. Further inquiries can be directed to the corresponding authors.

## Supplementary Materials

The following supporting information can be downloaded at: https://www.mdpi.com/article/10.3390/foods15111876/s1, Table S1: Summary of RNA sequencing quality control metrics for all samples.; Table S2: Statistics of read alignment to the reference genome.
